# Confab - Systematic generation of diverse low-energy conformers

**DOI:** 10.1186/1758-2946-3-8

**Published:** 2011-03-16

**Authors:** Noel M O'Boyle, Tim Vandermeersch, Christopher J Flynn, Anita R Maguire, Geoffrey R Hutchison

**Affiliations:** 1 Analytical and Biological Chemistry Research Facility, University College Cork, Western Road, Cork, Co. Cork, Ireland; 2 Open Babel development team; 3 Department of Chemistry, University of Pittsburgh, Chevron Science Center, 219 Parkman Avenue, Pittsburgh, PA 15260, USA

## Abstract

**Background:**

Many computational chemistry analyses require the generation of conformers, either on-the-fly, or in advance. We present Confab, an open source command-line application for the systematic generation of low-energy conformers according to a diversity criterion.

**Results:**

Confab generates conformations using the 'torsion driving approach' which involves iterating systematically through a set of allowed torsion angles for each rotatable bond. Energy is assessed using the MMFF94 forcefield. Diversity is measured using the heavy-atom root-mean-square deviation (RMSD) relative to conformers already stored. We investigated the recovery of crystal structures for a dataset of 1000 ligands from the Protein Data Bank with fewer than 1 million conformations. Confab can recover 97% of the molecules to within 1.5 Å at a diversity level of 1.5 Å and an energy cutoff of 50 kcal/mol.

**Conclusions:**

Confab is available from http://confab.googlecode.com.

## Introduction

The generation of molecular conformations is an essential part of many computational analyses in chemistry, particularly in the field of computational drug design. Methods such as 3D QSAR, protein-ligand docking and pharmacophore generation and searching [1] all require the generation of conformers, whether on-the-fly (as part of the method) or pre-generated by a stand-alone conformer generator. In contrast to 3D structure generators (such as CORINA [2], DG-AMMOS [3] and smi23d [4]), which focus on the generation of a single low-energy conformation, conformation generators create an ensemble of conformers that cover the entire space of low-energy conformations or that part of conformational space occupied by biologically-relevant conformers.

Several proprietary conformation generators are currently available (including OMEGA [5], ROTATE [6], Catalyst [7], Confort [8], ConfGen [9], Balloon [10] and MED-3DMC [11] among others) but only recently have open source conformation generators appeared: Frog2 [12] generates conformers using a Monte Carlo approach, while Multiconf-DOCK [13] adapts the systematic search code from DOCK5 [14] to generate diverse conformers via a torsion-driving approach.

Confab 1.0 is the first release of Confab, an open source conformation generator whose goal is the systematic coverage of conformational space. Accuracy has been favoured over the introduction of approximations to improve performance. The algorithm starts with an input 3D structure which, after some initialisation steps, is used to generate multiple conformers which are filtered on-the-fly to identify diverse low energy conformers. Conformations are generated using the torsion-driving approach from a set of predefined allowed torsion angles. Ring conformations are not currently sampled.

The first section of the paper describes the algorithm used by the software and some implementation details. After this, two applications of the software are described: an analysis of the conformational space of a dataset of 1000 molecules (which includes a comparison to Multiconf-DOCK), and an investigation of the conformational preferences of a particular phenyl sulfone.

## Methods

### Algorithm

**Figure 1:**
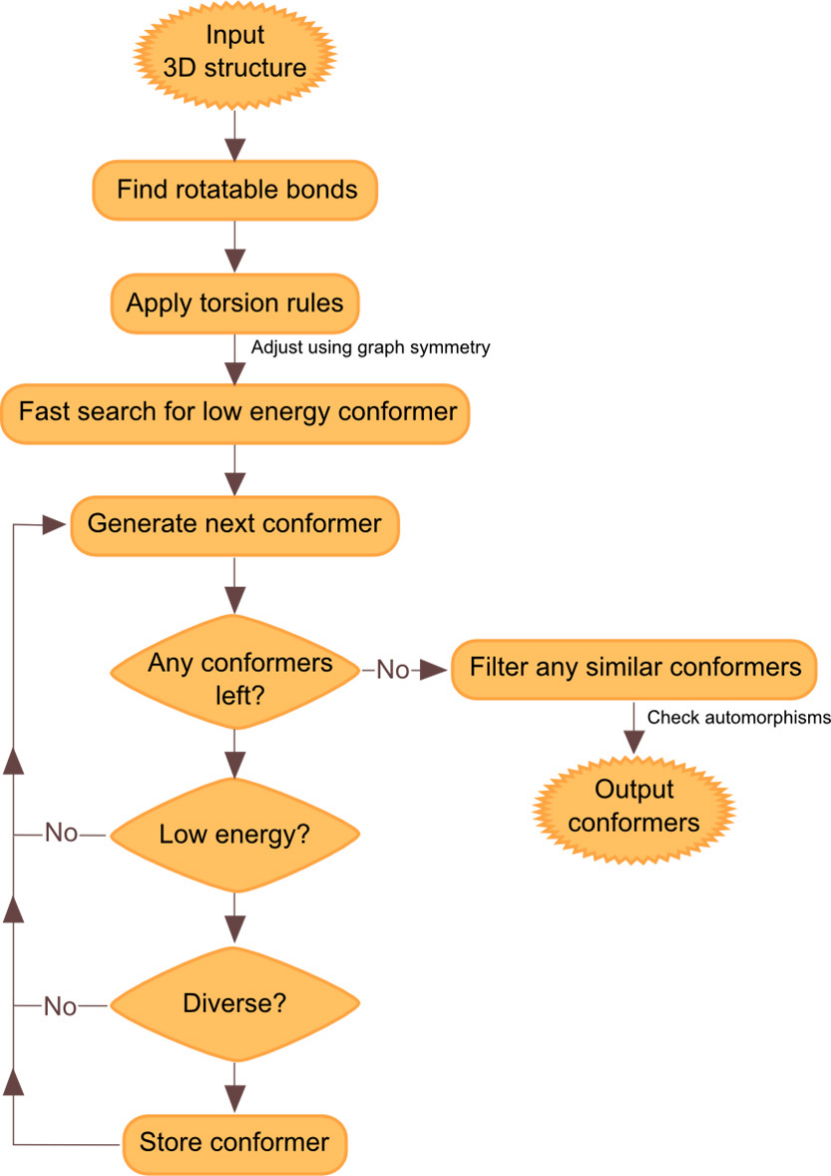
**Flowchart depicting the Confab algorithm**.

The Confab algorithm is outlined in Figure 1. The input required is a 3D structure with reasonable bond lengths and angles. Since the algorithm does not currently explore ring conformations, any rings present should be in reasonable conformations.

The first step of the algorithm is the identification of rotatable bonds. These are defined as all acyclic single bonds where both atoms of the bond are connected to at least two non-hydrogen atoms, but neither atom of the bond is sp-hybridised. Note that this definition excludes rotation around bonds that interchange hydrogens (for example, the rotation of the hydrogens of a methyl group), but this does not imply any loss of accuracy as it is usual practice to exclude hydrogens when calculating the RMSD (see below).

The method used by Confab to generate conformations is known as the torsion-driving approach. A set of allowed torsion angles for each rotatable bond is assigned to each bond by searching for a match to predefined SMARTS strings in a user-configurable file (torlib.txt) included in the Confab distribution. This file is part of the Open Babel project and it assigns values to particular rotatable bonds using data from Huang et al. [15].

Once the allowed torsion angles are assigned, they are corrected for topological (that is, graph) symmetry. The presence of such symmetry allows performance to be improved by eliminating redundant evaluations, thus reducing the number of conformations that will be tested. 2-fold symmetry is identified when a rotatable bond involves an sp^2 ^hybridised carbon atom where the neighbouring two atoms affected by the rotation are both of the same symmetry class. When this occurs the allowed values of that torsion are halved by restricting them to those less than 180°. The same is done for the case of 3-fold symmetry at an sp^3 ^hybridised carbon where the three neighbours are of the same symmetry class; in this case the torsion angles are restricted to those less than 120°. If graph symmetry is identified at both ends of a rotatable bond, the result is multiplicative; a 2-fold and a 3-fold symmetry combine to restrict allowed values of the torsion angles to 360/6 = 60°.

The next step is to obtain an estimate of the energy of the most stable conformer. Throughout Confab, energies are calculated using the MMFF94 forcefield [16]. The values of the bond stretching, angle bend, stretch bend and out-of-plane bending terms are constant for all conformers of the same molecule; only the torsion, Van der Waals and electrostatic terms were repeatedly evaluated. A low energy conformer is found using a simple greedy algorithm. Each torsion angle is optimised starting with the most central torsion and proceeding outwards. As this procedure is relatively fast (compared to the combinatorial problem of searching for the global optimum) it is repeated up to 16 times by testing the four most central torsions in different orders. The lowest energy conformer found is used as a reference point for applying an energy cutoff during the conformer search. If, during the actual conformer generation a lower energy conformer is found, this lower energy is used instead for the reference from that point on.

The main part of the algorithm is the systematic generation and assessment of all conformers described by the allowed torsion angles. Confab generates each of these in turn up to a user-specified cutoff (the default is 10^6^) and determines its energy relative to the lowest energy conformer found so far. If this is within a user-specified energy cutoff (50 kcal/mol by default), it is assessed for diversity to the conformers already stored (see below). If it is found to be diverse, it is itself stored otherwise it is discarded. The algorithm then moves onto the next conformer.

Rather than iterate in a 'depth-first' manner over the torsions and their allowed angles, Confab uses a Linear Feedback Shift Register (LFSR) to iterate in a random order over all of the conformers. A LFSR allows the generation of all integers from 1 to N pseudorandomly without repetition and without any memory overhead (which is important for large values of N). By iterating randomly, Confab avoids biasing generated conformers towards a particular region of conformational space, for example towards the input conformation. It also helps increase diversity if the number of possible conformations is greater than the cutoff for the number tested.

**Figure 2:**
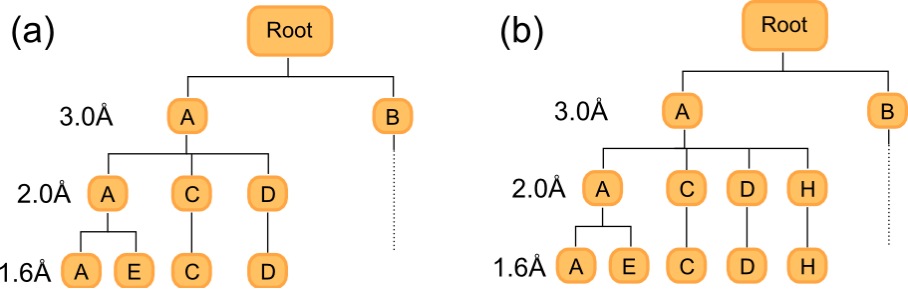
**An example diversity tree used to filter conformations on-the-fly**. (a) A diversity tree containing five conformations (A to E) used to filter conformations with an RMSD of less than 1.6 Å to one of the stored conformations. (b) The same diversity tree after addition of conformer H, where H is within 3.0 Å of A but not within 2.0 Å of A, C or D.

Diversity is ensured by calculating the heavy-atom RMSD (after least-squares alignment) of the newly generated conformation to those previously stored. The alignment is carried out using the QCP algorithm of Theobald [17] (which we found to be about twice as fast as the popular Kabsch alignment method [18]). Despite this, when a molecule has many conformers and a large number of conformers have been stored, full pairwise RMSD calculations take an excessive amount of time. To minimise the number of RMSD evaluations required to discard a conformer, chosen conformers are stored in a tree structure that effectively clusters conformers on-the-fly by RMSD. Figure 2(a) shows a typical 'diversity tree' where each level of the tree is associated with a smaller RMSD diversity from 3.0 Å down to the cutoff specified by the user (1.6 Å in the figure). Each node of the tree represents a stored conformation. Sibling nodes (that is, nodes at the same level that share the same parent node) differ by at least the RMSD diversity associated with that level. Note that sibling nodes are ordered and that the first child node of each parent is the same as the parent itself.

To illustrate the algorithm, let us imagine adding a new conformation H to the tree depicted in Figure 2(a). The algorithm starts at the top of the tree and determines which of the two branches (A or B) to take at the 3.0 Å diversity level. To do so it checks whether H is within 3.0 Å RMSD of A. If so, it follows the tree down to the next level, and checks to see whether it is within 2.0 Å RMSD of A (note that it does not need to recalculate the RMSD to do this). If this is not true, then it checks for 2.0 Å similarity to C. If so, it follows C down to the next level; otherwise it checks against D. If it is not similar to D, H is stored in the tree as the next sibling at that level of the tree (this is depicted in Figure 2(b)). When adding a new node for a conformation at a particular level, if the level is not at the bottom then child nodes containing that conformation are added at successively lower levels until the bottom level is reached. Overall, there are two possibilities; either the algorithm reaches the bottom level and finds that the new conformation is within the RMSD cutoff of an existing conformer, in which case it is discarded, or else it is of sufficient diversity to be stored at some level of the tree.

This algorithm greatly reduces the number of RMSD evaluations during the conformer generation loop. However it does not eliminate all conformations that are similar to those already stored; conformations may be retained that differ by less than the RMSD cutoff if they end up in different branches. To prune the set of retained conformations, while still avoiding a computationally expensive pairwise RMSD calculation, all of the retained conformations are added one-by-one to a new tree in order of increasing energy. This time the algorithm used for adding conformations to the diversity tree is more robust: all sibling conformations are tested for similarity, even after finding one that is similar. The result is that the same conformation may be added at several different points in the tree. This makes the tree more effective at eliminating similar conformations at the expense of a greater number of RMSD calculations.

Calculation of an RMSD can be overestimated when a molecule's structure has automorphisms (a permutation of the atoms of a molecule that preserves the bond connections). For example, if you consider a para-substituted phenyl ring where two conformations differ by a rotation of 180° around the substituted carbons, it is clear that the calculated RMSD between the conformations should be 0. However, if the symmetry of the phenyl ring is not taken into account this will not be the case and the RMSD will be overestimated as the corresponding atoms of the two structures have moved. The symmetry-corrected RMSD is obtained by iterating over the automorphisms of the molecule and taking the minimum value of the resulting RMSDs. For performance reasons, the calculation of the RMSD is not symmetry-corrected during the main conformation generation loop. However it is used afterwards when building the final diversity tree, thereby eliminating any conformations that were retained in error.

### Implementation

Confab is essentially a modified version of Open Babel [19], a widely-used cheminformatics toolkit written in C++ and available under the open source GPL v2 licence [20]. In fact, some of the code written for Confab has been merged into the main Open Babel distribution (such as the original Kabsch alignment code) but due to an additional dependency (on tree.hh, see below) the core code has not been included in Open Babel v2.3.

The MMFF94 forcefield, the conformer generation framework and the automorphism detection are all provided by Open Babel. QCP alignment was implemented using Theobald's public domain code [21] in combination with the Eigen2 high performance linear algebra library [22]. The diversity analysis code relies on a tree data structure provided by the Open Source tree.hh library [23]. The code used to implement the Linear Feedback Shift Register (LFSR) was adapted from its corresponding Wikipedia article [24]. Tap values for the register were taken from Alfke's Xilinx application note [25].

The Confab distribution contains two command-line applications: *confab *and *calcrmsd*. The former implements the Confab algorithm to generate conformers given an input 3D structure, while the latter may be used to assess the performance of *confab *by comparing the generated conformers to a file containing crystal structures. Full details of these applications are available on the Confab website.

## Coverage of Conformational Space

### Dataset

To illustrate the performance of Confab, we used a dataset of 1000 small molecule crystal structures derived from that of Borodina et al. [26]. The original source is the PDB; thus this dataset represents bioactive conformations of molecules. The 3D structures of the 14504 ligands in the Borodina dataset were obtained using the PubChem Download Service (using the PubChem Substance IDs from Borodina et al.). Of these, 16 could not be handled by the MMFF94 forcefield, 5202 had no rotatable bonds (this fraction included a large number of trivial salts) and 2348 had more than 1 million conformers (according to Confab's torsion rules). 1000 structures were randomly chosen from the 6938 remaining. See Additional file 1 for the structures of these 1000 molecules.

To avoid bias towards the crystal structures, the input conformations for Confab were generated by building the 3D structure using Open Babel. After the initial structure generation, the structures were optimised using the MMFF94 forcefield (200 steps steepest descent). Since Confab does not explore ring conformations, ring conformations were taken from the crystal structure for the initial structure generation. See Additional file 2 for the generated structures.

### Results

**Figure 3:**
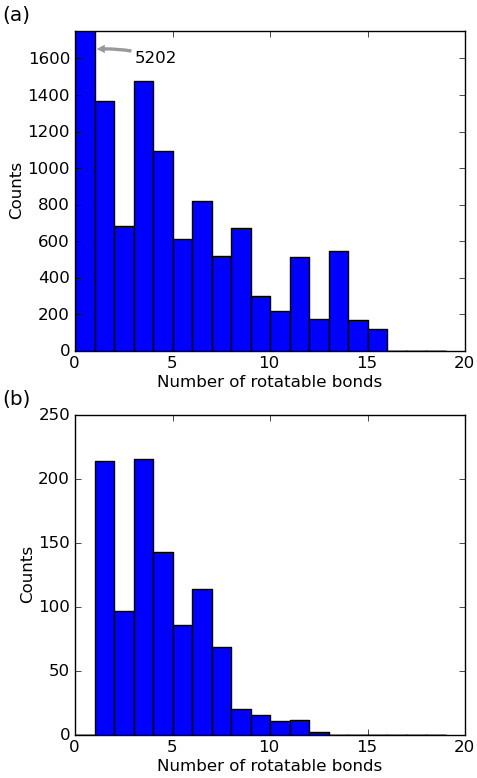
**The distribution of molecules in terms of the number of rotatable bonds in (a) the dataset of Borodina et al., and (b) our dataset of 1000 molecules**.

Figure 3(b) shows an overview of the dataset of 1000 structures in terms of the number of rotatable bonds in each molecule. Although the dataset contains molecules with up to 12 rotatable bonds, it is clear by comparison with the full dataset of Borodina et al. in Figure 3(a) that the reduced dataset is only a representative sample for molecules having up to 7 rotatable bonds. Beyond this, the restriction that the molecule must have fewer than 1 million conformers leads to the elimination of most of the molecules. For this reason, to avoid erroneous conclusions some of the following analyses (where stated) will not consider molecules having 8 or more rotatable bonds.

**Figure 4:**
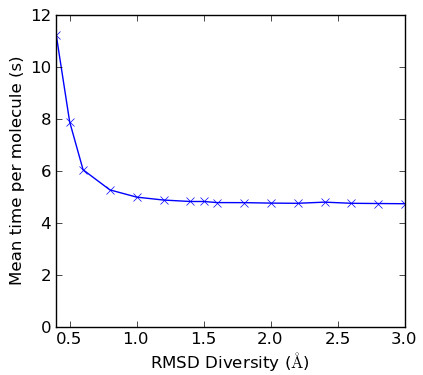
**Effect of diversity level on speed of conformer generation**. Times were measured on an Intel Xeon E5620 Processor (2.4GHz, 4C) with 32GB RAM.

Confab was used to exhaustively generate all low energy conformers for each molecule in the dataset for diversity values ranging from 0.4 Å to 3.0 Å RMSD. The default setting of 50 kcal/mol was used as an energy cutoff. The default value of 1 million conformers was used as the conformer cutoff; this ensured exhaustive coverage of conformational space (as defined by Confab's torsion rules) as structures with more conformers were not included in the dataset (see above). Figure 4 shows the mean time for conformer generation per molecule. This is largely independent of the diversity level for diversity levels greater than or equal to 1.0 Å. For values less than this, an increasing amount of time is spent performing the pairwise RMSD calculations against stored conformations.

Performance of conformer generators is typically measured by the percent recovery of crystal structures with respect to a particular RMSD cutoff (see for example Ref [9]). This is simply the percentage of molecules which have a generated conformer within a particular RMSD of the crystal structure. Commonly used values for this RMSD cutoff are 2.0, 1.5 and 1.0 Å.

**Figure 5:**
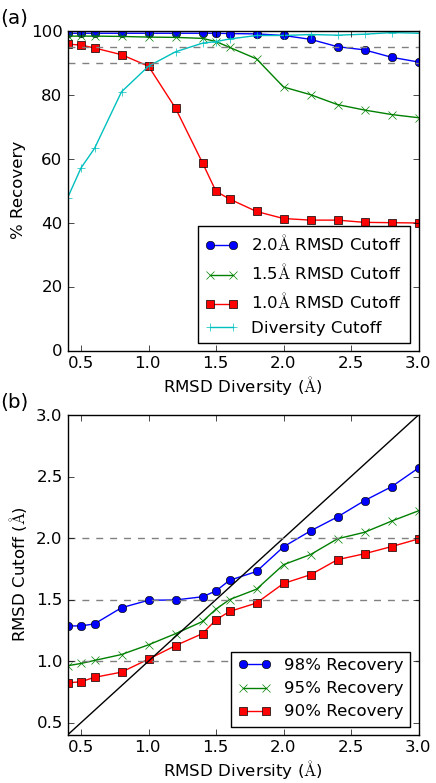
**Performance measured as % recovery of crystal structures**. (a) Performance for different RMSD cutoffs. The diversity cutoff is where the value of the RMSD diversity is used as the RMSD cutoff. (b) The RMSD cutoff required to achieve a particular level of % recovery. The diagonal line indicates the maximum RMSD cutoff expected when there is complete coverage.

Figure 5(a) shows the percent recovery at these cutoffs for different values of the RMSD diversity. At 2.0 Å RMSD diversity, 99% are within 2.0 Å RMSD of the crystal (83% within 1.5, 41% within 1.0); at 1.5 Å RMSD diversity, 99% are within 2.0 Å (97% within 1.5, 50% within 1.0); at 1.0 Å RMSD diversity, 99% are within 2.0 Å RMSD (98% within 1.5, 89% within 1.0). As expected, the percentage of crystal structures that are found decreases as the RMSD diversity increases. In particular, the curves fall off steeply once the RMSD diversity is greater than the required cutoff.

An interesting question to ask is what RMSD diversity is required to recover X% of crystal structures with respect to a particular RMSD cutoff? Figure 5(b) shows the answer to this where X is 90%, 95% or 98%. For example to find 95% of the crystal structures within a 2.0 Å cutoff an RMSD diversity of 2.4 Å (or smaller) is required, but to find the same percentage to within 1.5 Å an RMSD diversity of 1.6 Å is needed. However, even an RMSD diversity of 0.4 Å will not recover 98% of the structures to within 1.0 Å (it only recovers 96%), an indication of the inherent diversity of the generated conformers as discussed further below.

As pointed out by Borodina et al. [26], if the conformational space is perfectly covered and lacks any 'holes' then the RMSD diversity is an upper bound of the minimum RMSD to the crystal structure. In other words, at an RMSD diversity of 1.5 Å for example, all crystal structures should be found to within 1.5 Å. The diagonal line in Figure 5(b) indicates the maximum RMSD cutoff expected if this ideal behaviour is observed. It is clear from the figure that at low RMSD diversity the actual performance is poorer than this.

There are two main problems that give rise to gaps in conformational coverage. The first is that the allowed torsion values may not encompass the specific torsion angle observed in the crystal structure. For this dataset, there are 7 molecules for which the crystal structure could not be found within 2.0 Å even at 0.4 Å RMSD diversity. These molecules (PubChem substance IDs of 584680, 823881, 825747, 826196, 828032, 830919 and 834618), of which two represent different conformations of the same molecule, all involve sugar moieties and it may be that the allowed torsion angles of the glycosidic bond are too conservative.

The second is that the granularity of the allowed torsion settings may not be sufficiently fine to allow solutions to be found to within a low RMSD cutoff. For example, a carbon-carbon single bond has 12 allowed torsion values from 0 to 360° in increments of 30°. If such a bond is centrally located in a large molecule, even if the crystal structure has similar torsion angles to one of these conformers the RMSD may differ significantly.

Based on this dataset, the inherent granularity of the Confab generated conformers is around 1.4 Å, as indicated by the "Diversity Cutoff" line in Figure 5(a) which falls off sharply as the RMSD diversity decreases below 1.4 Å. This line indicates the percent recovery at different levels of RMSD diversity when the RMSD cutoff used is the same as the diversity level. The sharp fall off below 1.4 Å is a deviation from the ideal behaviour described by Borodina et al.

Table 1 shows the median number of generated conformers tested for molecules with different numbers of rotatable bonds. Broadly speaking, about one third of the conformers pass the energy cutoff applied. Although the size of each individual subset is not very large, and the values for 6 rotatable bonds seem to be biased towards a larger number of conformers, some general points can still be made.

**Table 1:** Relationship between the number of rotatable bonds, the number of conformers generated and the minimum RMSD to the crystal structure.

Rotatable bonds^†^	Number of molecules	Total Conformers (median)	Low Energy Conformers (median)	Diverse Conformers (median)					Minimum RMSD to crystal (median)				
				
				0.5 Å	1.0 Å	1.5 Å	2.0 Å	3.0 Å	0.5 Å	1.0 Å	1.5 Å	2.0 Å	3.0 Å
1	214	3	3	3	1	1	1	1	0.18	0.40	0.45	0.45	0.45

2	97	36	25	8	2	1	1	1	0.34	0.54	0.74	0.80	0.80

3	216	72	44	19	4	1	1	1	0.39	0.70	1.02	1.06	1.06

4	143	1296	582	96	9	2	1	1	0.52	0.80	1.07	1.14	1.24

5	86	3024	1065	189	24	4	1	1	0.60	0.82	1.14	1.31	1.34

6	114	186624	24317	2953	192	24	5	1	0.71	0.90	1.21	1.49	1.78

7	69	34992	10679	1402	139	17	4	1	0.66	0.83	1.14	1.44	1.73

^†^The 61 molecules with 8 or more rotatable bonds are omitted.

The number of diverse conformers is much reduced by a higher diversity level. For example, for those molecules with 7 rotatable bonds there are approximately 11000 low energy conformers of which about 13% are diverse at 0.5 Å RMSD, only 1.3% are diverse at 1.0 Å RMSD, and only 0.16% are diverse at 1.5 Å RMSD.

The values in Table 1 are in broad agreement with those reported by Smellie et al. [27] for a representative subset of their dataset (see table three therein). They make the point that the number of conformers required to cover conformational space is really surprisingly low. For a molecule with 7 rotatable bonds in our dataset, conformational space can be covered to within 1.0 Å with merely hundreds of conformations while just tens of conformations will achieve a coverage of 1.5 Å. Of course, these figures are expected to increase with each additional rotatable bond.

For completeness, Table 1 also reports median values for the minimum RMSD to the crystal structure. However, as a metric for coverage these values give a misleadingly positive picture compared to the percent recovery values discussed above.

### Comparison with Multiconf-DOCK

Multiconf-DOCK [13] is another open source conformer generator that uses a torsion driving approach to implement a systematic search to identify diverse low energy conformers. This software differs in that it uses the AMBER force field [28,29] (as implemented in DOCK5) instead of MMFF94. In addition, it implements performance improvements such as search tree pruning by partial energy estimation [14]. Like Confab, the software requires a 3D structure as input.

Multiconf-DOCK was used to generate conformations for the 1000 structures in the dataset using the same input as for Confab but converted to MOL2 using Open Babel v2.3.0. It should be noted that the specified Sybyl atom types in the input MOL2 file have an effect on the conformations generated by Multiconf-DOCK. The parameters used were taken from the example provided with the Multiconf-DOCK distribution, except that no restriction was placed on the number of generated conformations and the energy cutoff was set to 50 kcal/mol (as used for Confab). Three different RMSD diversity levels were investigated: 2.0 Å, 1.5 Å and 1.0 Å. For all three diversity levels, the mean time spent per molecule was 6.3 s (measured on the same machine used for Figure 4).

The performance in terms of percent recovery is as follows: at 2.0 Å RMSD diversity, 99% are within 2.0 Å RMSD of the crystal structure (89% within 1.5, 55% within 1.0); at 1.5 Å RMSD diversity, 99% are within 2.0 Å (97% within 1.5, 64% within 1.0); at 1.0 Å RMSD diversity, 99% are within 2.0 Å (98% within 1.5, 80% within 1.0). These values are broadly similar to those for Confab (see above). The most noticeable differences occur for the percentage of structures found to within 1.0 Å RMSD; assuming that both programs successfully remove conformations that are within the diversity cutoff, Multiconf-DOCK outperforms Confab at the 2.0 Å and 1.5 Å RMSD diversity levels but Confab performs better at 1.0 Å RMSD diversity.

Table 2 shows the median number of conformers generated by Multiconf-DOCK, along with the minimum RMSD to the crystal structure, broken down by the number of rotatable bonds. Compared to Confab the number of conformers generated is far fewer. It is difficult to say whether this represents a less comprehensive coverage of conformational space or whether this is due to the use of different forcefields. In terms of the minimum RMSD to the crystal structure, once again we see that Multiconf-DOCK performs better than Confab at the 2.0 Å and 1.5 Å RMSD diversity levels but Confab is better at 1.0 Å RMSD diversity.

**Table 2:** Results for Multiconf-DOCK showing the relationship between the number of rotatable bonds, the number of conformers generated and the minimum RMSD to the crystal structure.

Rotatable bonds	Diverse Conformers (median)			Minimum RMSD to crystal (median)		
	
	1.0 Å	1.5 Å	2.0 Å	1.0 Å	1.5 Å	2.0 Å
1	1	1	1	0.34	0.40	0.40

2	3	1	1	0.50	0.67	0.71

3	2	1	1	0.68	0.78	0.81

4	9	3	1	0.76	0.97	1.05

5	14	4	2	0.85	1.03	1.28

6	43	15	5	1.08	1.23	1.37

7	21	8	3	1.04	1.24	1.40

## Distance Distribution in Conformations of a Phenyl Sulfone

**Figure 6:**
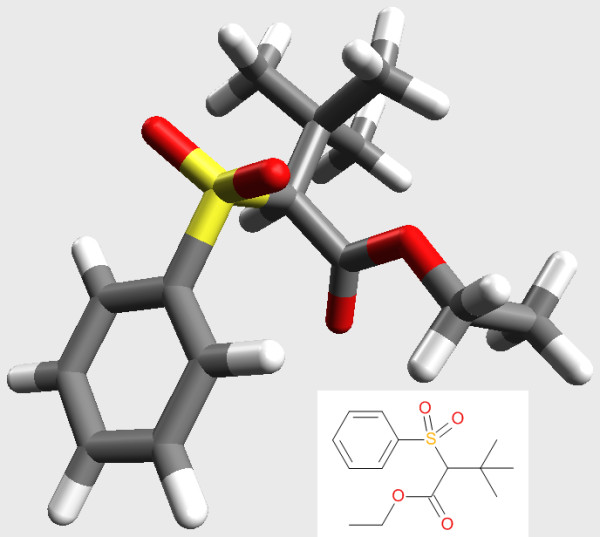
**Structure of the phenyl sulfone studied**.

Many conformer generators are focused on reproducing bioactive conformations. However it is worth remembering that the generation of conformers may also be useful in other contexts. Here we use Confab to as an aid to interpret the NMR spectra for the phenyl sulfone shown in Figure 6. The peak for the methylene carbon of the ethyl ester was split unexpectedly (compared to an analogous sulfone where the phenyl group was replaced by tert-butyl), and our hypothesis was that this was due to the close approach of the methylene carbon to one of the sulfonyl oxygens in solution. Confab was used to investigate whether low energy conformations existed where the methylene group was in close proximity to a sulfonyl oxygen.

Confab was used to generate a set of conformations of the molecule with a diversity of 0.2 Å and no energy cutoff. The resulting 2014 conformations were optimised using a MMFF94 forcefield (200 steps steepest descent; implemented using Pybel [30]) and the final energy recorded. For each of the conformations the minimum distance between a sulfonyl oxygen and the methylene carbon was measured.

**Figure 7:**
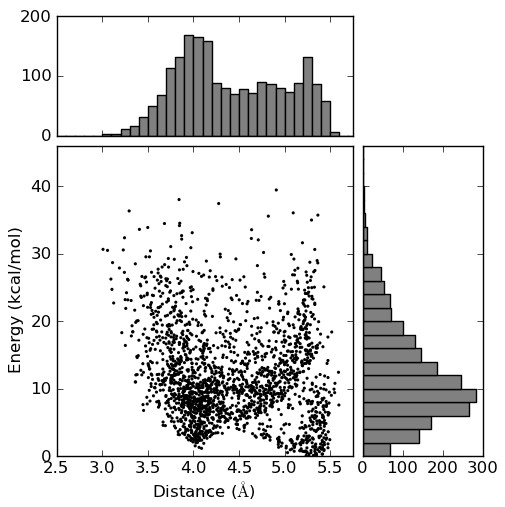
**Scatterplot with marginal histograms of distance versus energy for the set of conformations of the phenyl sulfone in Figure 6**.

Figure 7 shows a plot of these distances versus the relative energies of the conformers with marginal histograms showing the distribution of values. The methylene carbon does not approach the sulfonyl group very closely. For low energy conformers, the distances are clustered around 4.0 Å and 5.4 Å with the former more frequent. Taking 5 kcal/mol as a cutoff, the distance can be as low as 3.7 Å but shorter distances (down to 3.0 Å) are only possible with an associated energy penalty. Figure 6 shows one of the low energy conformations (relative energy of 4.6 kcal/mol) which has a distance of 3.7 Å between the groups of interest.

## Conclusion

The goal of this first release of Confab is to ensure complete coverage of all of the low energy conformers of a molecule. While every effort is made to maximise performance, accuracy has been the main goal. Approximations that reduce the search space on the basis of heuristics have been avoided for this reason.

Using the results from Confab 1.0 as a comparison, future work will investigate strategies to to overcome the combinatorial explosion associated with large numbers of rotatable bonds [31] including the trade-off between speed and accuracy.

## Availability and Requirements

**Project name: **Confab

**Project home page: **http://confab.googlecode.com

**Operating system(s): **Cross-platform

**Programming language: **C++

**Other requirements (if compiling): **CMake 2.4+, Eigen2

**Licence: **GPL v2

**Any restrictions to use by non-academics: **None

## Authors' contributions

NMOB devised and implemented Confab, and carried out the coverage analysis. GRH implemented the conformer generation framework in Open Babel and contributed to the forcefield code. TV implemented the automorphism code in Open Babel and contributed to the forcefield code. NMOB collaborated with CJF and ARM on the sulfone investigation. All authors read and approved the final manuscript.

## Supplementary Material

Additional file 1 **Crystal structures used to test conformational coverage**. This is a text file in SDF format containing biological conformations (as downloaded from PubChem) of 1000 molecules. This is a subset of the data used in the study by Borodina et al. Click here for file

Additional file 2 **Generated 3D structures used to test conformational coverage**. This is a text file in SDF format containing 3D structures of the 1000 molecules in the dataset generated using Open Babel. These were used as the input to Confab. Click here for file
